# Editorial: Probiotics, prebiotics, synbiotics, postbiotics, & paraprobiotics - New perspective for functional foods and nutraceuticals

**DOI:** 10.3389/fnut.2023.1164676

**Published:** 2023-03-17

**Authors:** Reshma B Nambiar, Anand Babu Perumal, Taofik Shittu, Emmanuel Rotimi Sadiku, Periyar Selvam Sellamuthu

**Affiliations:** ^1^College of Animal Science, Zhejiang University, Hangzhou, China; ^2^College of Biosystems Engineering and Food Science, Zhejiang University, Hangzhou, China; ^3^Department of Food Science and Technology, College of Food Science and Human Ecology, Federal University of Agriculture, Abeokuta, Nigeria; ^4^Department of Chemical, Metallurgical and Materials Engineering, Institute of NanoEngineering Research, Tshwane University of Technology, Pretoria, South Africa; ^5^Department of Food Process Engineering, Postharvest Research Lab, School of Bioengineering, SRM Institute of Science and Technology, Kattankulathur, Tamilnadu, India

**Keywords:** food chemistry, functional foods, nutraceuticals, nanodelivery systems, packaging

Probiotics are “live microorganisms which when administered in adequate amount confer health benefits on the host” (1). These health benefits include inhibiting gastrointestinal pathogens by modifying their immunogenicity and enhancing the degradation of enteric antigens and improving barrier protection, lactose intolerance, or diarrhea, inflammatory bowel disease, and anti-mutagenic activity. The safety of probiotics should be taken into account when administrated to the elderly and immunodeficient individuals. In order to protect the viability of probiotics, it is recommended to use carrier materials and prebiotics, such as maltodextrin, fructooligosaccharide, and inulin (2, 3).

A new perspective on functional foods and nutraceuticals concepts aims to improve the viability of probiotics with new technological improvements (3–6). The main aim of this Research Topic for “Frontiers in Nutrition” is to provide an overview of the central issues on probiotic-related functional foods and pharmaceutical products and to highlight and address the urgent need for further studies, aimed at evaluating the safety and efficacy of these products and their mechanisms of action. It aims to highlight the recent progress made toward developing pre- to postbiotics-related products for boosting health and immune systems.

This Research Topic is comprised of eight articles (including and reviews), contributed to by 42 authors.

Diarrhea is the most common adverse reaction in antibiotic treatment, and is generally initiated by the imbalance of intestinal flora; probiotics, which play a vital role in balancing intestinal flora diversity, can be useful in addressing this. Bao et al. showed that gastric perfusion of the strain *Lactiplantibacillus plantarum* 2-33 remains in the intestinal flora of antibiotic-associated diarrhea (AAD) mice for up to 7 days, but the relative abundance and diversity of intestinal flora of AAD mice were significantly (*p* < 0.05) improved by gastric perfusion for 14 days. Moreover, strain 2-33 exhibited a time- and dose-dependent effect on the intestinal flora of AAD mice, whereas short-term gastric perfusion and low dose had no significant effect (*p* > 0.05). The study showed that strain 2-33 enhanced the structure and diversity of the intestinal flora of AAD mice, balanced the level of substance and energy metabolism, and played an important role in curing diarrhea, maintaining and increasing the intestinal microecological balance.

Probiotic bacteria have potential uses as immunomodulators. However, comparative data on their immunological effects are very limited. The study by Fong et al. aimed to characterize the effect of an oral administration of the commonly used probiotic strains, alone or as mixtures, on the systemic and organ-specific immune responses of C57BL/6 mice. The study confirmed that the immunomodulatory potentials, such as cytokine production and cell-mediated responses of the probiotics, is strain- and organ/tissue-specific, and the effects of probiotic mixtures cannot be predicted based on their single constituents.

Bacteriocins are able to impede the growth of intestinal pathogenic bacteria and modulate the gut microbiota in animals. However, there is little knowledge on their effect on gut microbiota of different enterotypes of human. Pu et al. evaluated the modification of the gut microbiota of two enterotypes (ET B and ET P) by the class IIb bacteriocin plantaricin NC8 (PLNC8) by using an *in-vitro* fermentation model of the intestine. Gas chromatography results revealed that PLNC8 had no influence on the gut microbiota's production of short-chain fatty acids in the subjects' samples. PLNC8 lowered the Shannon index of ET B' gut microbiota and the Simpson index of ET P' gut microbiota, based on 16S rDNA sequencing. Cheng et al. reviewed the role of *Bifidobacterium animalis* subsp. *lactis* HN019 (*B. lactis* HN019TM) on gut health and its mechanisms of action in several preclinical and clinical studies. *B. lactis* HN019TM has a beneficial role in maintaining intestinal barrier function during gastrointestinal infections by competing and excluding potential pathogens *via* different mechanisms, maintaining normal tight junction function *in-vitro* and regulating the host immune defense toward pathogens in both the *in-vitro* and human studies. In addition, *B. lactis* HN019TM reduced the intestinal transit time and increased the bowel movement frequency in functional constipation by modulating the gut-brain-microbiota axis, mainly *via* the serotonin signaling pathway and through short chain fatty acids, derived from microbial fermentation.

Recent studies have reported that tender coconut water and rice has the same effect as a prebiotic (3). The prebiotic activities of Riceberry rice (R), germinated Riceberry rice (GR), and germinated Riceberry rice with mycelium (GRM) were assessed on the probiotic bacteria *Pediococcus* sp., *Lactobacillus acidophilus*, and *Streptococcus lactis* (Soodpakdee et al.). Results revealed the enhancement of prebiotic properties on GR, although R did not indicate any prebiotic activity in any probiotic bacteria. GR exhibited moderate prebiotic properties on *Pediococcus* sp., *L. acidophilus*, and *S. lactis*. Moreover, the prebiotic properties of GR were enhanced when fermented with *Pleurotus ostreatus* mycelium (M). The study showed that GRM could be used on rice-based products to enhance their nutritional value and improve digestive system health, particularly in the elderly. Sun et al. provided novel insights into the bifidogenic and antibacterial activities of xylooligosaccharides (XOS). Three growth indicators showed strain-specific bifidogenic activity of XOS, and the activity was both dosage- and fraction-dependent, since only certain fractions stimulated significant growth.

There are multiple applications of probiotics. Interestingly, Hashemi et al. revealed an insight into the potential application of synbiotic edible films and coatings in food products. Numerous bioactive compounds, such as antioxidants, antimicrobials, flavoring agents, colors, probiotics, and prebiotics, could be incorporated into films and coatings (7, 8). The incorporation of probiotics into edible films and coatings is an alternative approach for direct application in food matrices that enhances their stability and functional properties (9). In recent times, the addition of probiotics, along with prebiotic compounds such as inulin, starch, fructooligosaccharide, polydextrose, and wheat dextrin, has emerged as a new form of bioactive packaging. The simultaneous application of probiotics and prebiotics has improved the viability of probiotic strains, elevated their colonization in the intestinal tract, and provided health benefits to humans (10). Koyum et al. used five probiotic strains to establish the fact that staple foods, produced from composite flours, are able to alleviate protein-energy malnutrition. The biotransformation process, mediated by probiotics *via* the solid-state fermentation (SSF) process, has potential to address the poor protein digestibility in composite flours. The study showed that there was effective biotransformation of gluten-free composite flour, mediated by probiotics, *via* SSF and the flour quality was better at lower moisture content.

In conclusion, this Research Topic not only discusses the fundamental knowledge of prebiotics, probiotics, and synbiotics, their unique properties, and multipurpose applications in different arenas, but also provides the bases for the development of functional foods and nutraceuticals, based on innovative resources for novel and emerging applications and discusses the pilot-scale production and commercialization of biotics/pre-pro-synbiotics-based products.

## Author contributions

RN and AP wrote the editorial with input from TS, ES, and PS. All authors contributed to the article and approved the submitted version.
